# Impact of flat chest on waitlist mortality in adult patients with pleuroparenchymal fibroelastosis listed for lung transplantation

**DOI:** 10.1016/j.jhlto.2026.100509

**Published:** 2026-02-06

**Authors:** Chihiro Konoeda, Gouji Toyokawa, Miho Yamaguchi, Takafumi Yamaya, Takaki Akamine, Mitsuaki Kawashima, Mototsugu Shimokawa, Masaaki Sato

**Affiliations:** a Department of Thoracic Surgery, The University of Tokyo Hospital, Tokyo, Japan; b Department of Surgery and Science, Graduate School of Medical Sciences, Kyushu University, Fukuoka, Japan; c Department of Biostatistics, Graduate School of Medicine, Yamaguchi University, Yamaguchi, Japan

**Keywords:** Pleuroparenchymal fibroelastosis, Flat chest, APDT/TDT, Lung transplantation, Waitlist mortality

## Abstract

**Background:**

Pleuroparenchymal fibroelastosis (PPFE) is a distinct subtype of idiopathic interstitial pneumonia, and progressive cases require lung transplantation (LT). Some patients with PPFE develop chest flattening; however, its impact on waitlist mortality for LT remains unclear.

**Methods:**

We retrospectively analyzed adult patients with PPFE listed for LT from donation after brain death (DBD) between January 2014 and July 2024. Chest flatness was quantified as the ratio of the anteroposterior diameter of the thoracic cage to its transverse diameter (APDT/TDT). An APDT/TDT cut-off for waitlist mortality was determined by receiver operating characteristic curve analysis. The primary objective was to investigate the prognostic significance of APDT/TDT on waitlist mortality.

**Results:**

Among 36 listed patients, 19 (52.8%) underwent LT, 14 (38.9%) died while awaiting LT, and 3 (8.3%) remained on the waitlist. Patients were classified into high and low APDT/TDT groups: 11 (30.6%) and 25 (69.4%), respectively. The low APDT/TDT group demonstrated shorter 6-minute walk distance (6MWD; *P* = 0.002), lower percentage forced vital capacity (*P* = 0.015), and higher pCO_2_ (*P* = 0.035). Waitlist survival was shorter in the low APDT/TDT group than in the high group (*P* = 0.010). Univariate analyses identified low APDT/TDT (*P* = 0.033), smoking history (*P* = 0.012), and 6MWD < 400 m (*P* = 0.007) as risk factors for waitlist mortality.

**Conclusions:**

The APDT /TDT, an index of a flat chest, may serve as a prognostic factor for waitlist mortality in patients with PPFE awaiting LT.

## Introduction

Pleuroparenchymal fibroelastosis (PPFE) is a distinct subtype of idiopathic interstitial pneumonias (IIPs) characterized by upper‑lobe–predominant pulmonary fibrosis accompanied by fibrous thickening of the visceral pleura.^1^ Patients with PPFE exhibit several characteristic clinical features, including progressive weight loss, restrictive ventilatory impairment with increased residual volume, and recurrent pneumothorax.^2^, ^3^ As the disease progresses, some patients develop flattening of the thoracic cage,^4^ and a previous study demonstrated that the ratio of the anteroposterior diameter of the thoracic cage to its transverse diameter (APDT/TDT), an index of a flat chest, is significantly higher in patients with PPFE than in those with other IIPs.^5^ The therapeutic efficacy of steroid and antifibrotic agents is limited, and lung transplantation (LT) remains the only established treatment for advanced PPFE.^2^ However, not all patients with PPFE are able to undergo LT, as a substantial proportion die while awaiting LT. Identifying PPFE-specific factors that inform the optimal timing of referral to LT centers and listing for LT is therefore essential to reduce waitlist mortality and improve access to LT in this population.

In this study, the prognostic significance of APDT/TDT and other clinical factors for waitlist mortality was investigated among adult patients with PPFE awaiting LT from donation after brain death (DBD).

## Methods

### Study cohort

Between January 2014 and July 2024, 478 adult patients aged 18 years or older were listed for LT from DBD at The University of Tokyo Hospital. Among these patients, 253 were diagnosed with interstitial lung disease, including 36 with PPFE. Clinical data, including the ratio of the anteroposterior diameter of the thoracic cage to its transverse diameter (APDT/TDT), were retrospectively collected at the time of listing for LT from DBD through the Japan Organ Transplant Network. Information on LT from DBD, waiting time, and mortality were collected until the cut-off date in July 2025. The primary objective was to evaluate the prognostic significance of APDT/TDT and other clinical factors for waitlist mortality in adult patients with PPFE awaiting LT from DBD.

### Clinical data and measurement of APDT and TDT on computed tomography

Clinical and radiological data, including blood test results and APDT/TDT, were obtained at admission for evaluation of LT listing eligibility. APDT/TDT was measured in accordance with the method described by Harada et al., with measurements performed at the level of the sixth rib on axial computed tomography (CT) images acquired at the time of listing.^4^ In this method, APDT is defined as the longest anteroposterior dimension of the thoracic cage, measured perpendicular to a line drawn along the rearmost points of the sixth thoracic vertebra. The optimal APDT/TDT cut-off value for waitlist mortality was determined using receiver operating characteristic (ROC) curve analysis. The geriatric nutritional risk index (GNRI) was calculated as (1.489 x serum albumin [g/dL]) + (41.7 x [current body weight / ideal body weight]), and a cut-off value of 93.84 was applied based on previous work.^6^

### Statistical analysis

Categorical variables are presented as numbers and percentages, and continuous variables as medians with first and third interquartile ranges (IQRs). Overall survival (OS) was defined as the interval from listing for LT from DBD to death from any cause. Patients who remained on the waiting list at the cut-off date and those who underwent LT were censored. Associations between APDT/TDT and continuous variables were assessed using Student’s t-test for normally distributed data and the Mann–Whitney or Kruskal–Wallis test for non-normally distributed data. Categorical variables were compared using the chi-squared test. Survival probabilities were estimated using the Kaplan–Meier method and compared with the log-rank test. Hazard ratios (HRs) with 95% confidence intervals (CIs) were calculated using univariate analysis. Missing data were not complemented. A *P*-value < 0.05 was considered statistically significant. Analyses were performed using JMP® 18.0 (SAS Institute, Cary, NC, USA) and Prism 8.0 (GraphPad Software, San Diego, CA, USA).

## Results

### Patient characteristics

Baseline characteristics of the 36 patients with PPFE at the time of listing for LT from DBD are summarized in Table 1. Among these patients, 35 (97.2%) were classified as having idiopathic PPFE (IPPFE) and 1 (2.8%) as secondary PPFE occurring after bone marrow transplantation. The median age was 54 years (first, third IQR: 47, 57); 13 patients (36.1%) were female, and 16 (44.4%) had a history of smoking. Median body mass index and 6-minute walk distance (6MWD) were 17.0 kg/m^2^ (first, third IQR: 15.9, 19.6) and 410 m (first, third IQR: 311, 493), respectively. Fourteen patients (38.9%) had a high GNRI, whereas 22 (61.1%) had a low GNRI. Oxygen supplementation at rest was required in 5 patients (13.9%), and 2 (5.6%) had pulmonary hypertension. A history of pneumothorax was present in 15 patients (41.7%). Six patients (16.7%) and 8 (22.2%) had received steroids and antifibrotic agents, respectively. Median percentage forced vital capacity (%FVC) was 45.7% (first, third IQR: 31.4, 50.2), and median percentage diffusing capacity of the lung for carbon monoxide (%DLCO) was 60.5% (first, third IQR: 48.9%, 68.1%). Median partial pressures of oxygen and carbon dioxide (pCO_2_) were 83.3 mmHg (first, third IQR: 72.8, 89.1) and 43.8 mmHg (first, third IQR: 40.5, 48.0), respectively. During follow-up, 19 patients (52.8%) underwent LT, 3 (8.3%) remained on the waiting list, and 14 (38.9%) died while awaiting LT.

**Table 1:** Characteristics of PPFE patients listed for lung transplantation from donation after brain death and comparison of the characteristics between high and low APDT/TDT groups

Demographics		All (n = 36)	High APDT/TDT group (n = 11 [30.6%])	Low APDT/TDT group (n = 25 [69.4%])	*P-*value
Type of PPFE	Idiopathic	35 (97.2%)	11 (100.0%)	24 (96.0%)	0.501
	Secondary	1 (2.8%)	0 (0.0%)	1 (4.0%)	
Age (years), median (IQR)		54 (47, 57)	56 (52, 58)	54 (44, 56)	0.317
	≥ 55 years	21 (58.3%)	5 (45.5%)	16 (64.0%)	0.299
	<55 years	15 (41.7%)	6 (54.5%)	9 (36.0%)	
Sex	Female	13 (36.1%)	4 (36.4%)	9 (36.0%)	0.983
	Male	23 (63.9%)	7 (63.6%)	16 (64.0%)	
Smoking history	Never	20 (55.6%)	7 (63.6%)	13 (52.0%)	0.518
	Former	16 (44.4%)	4 (36.4%)	12 (48.0%)	
BMI (kg/m^2^), median (IQR)		17.0 (15.9, 19.6)	18.2 (16.6, 18.2)	16.7 (15.6, 18.3)	0.146
	≥ 20 kg/m^2^	8 (22.2%)	3 (27.3%)	5 (20.0%)	0.629
	< 20 kg/m^2^	28 (77.8%)	8 (72.7%)	20 (80.0%)	
6-min walk distance (m), median (IQR)		410 (311, 493)	515 (450, 645)	375 (306, 432)	0.002
	≥ 400 m	19 (52.8%)	10 (90.9%)	9 (36.0%)	0.002
	< 400 m	17 (47.2%)	1 (9.1%)	16 (64.0%)	
GNRI, median (IQR)		92.63 (86.84, 95.57)	94.57 (86.63, 102.91)	91.57 (86.92, 95.19)	0.265
GNRI	High (≥ 93.84)	14 (38.9%)	6 (54.6%)	8 (32.0%)	0.201
	Low (< 93.84)	22 (61.1%)	5 (45.4%)	17 (68.0%)	
Blood type	A	15 (41.7%)	4 (36.4%)	11 (44.0%)	0.960
	O	10 (27.8%)	3 (27.3%)	7 (28.0%)	
	B	8 (22.2%)	3 (27.3%)	5 (20.0%)	
	AB	3 (8.3%)	1 (9.0%)	2 (8.0%)	
Oxygen supplementation at rest	No	31 (86.1%)	11 (100.0%)	20 (80.0%)	0.110
	Yes	5 (13.9%)	0 (0.0%)	5 (20.0%)	
History of pneumothorax	No	21 (58.3%)	9 (81.8%)	12 (48.0%)	0.058
	Yes	15 (41.7%)	2 (18.2%)	13 (52.0%)	
Pulmonary hypertension	No	34 (9.4%)	11 (100.0%)	23 (92.0%)	0.334
	Yes	2 (5.6%)	0 (0.0%)	2 (8.0%)	
Use of steroid	No	30 (83.3%)	10 (90.9%)	20 (80.0%)	0.419
	Yes	6 (16.7%)	1 (9.1%)	5 (20.0%)	
Use of antifibrotic agents	No	28 (77.8%)	10 (90.9%)	18 (72.0%)	0.209
	Yes	8 (22.2%)	1 (9.1%)	7 (28.0%)	
%FVC (%), median (IQR)		45.7 (31.4, 50.2)	49.7 (46.0, 51.2)	36.9 (26.9, 48.8)	0.015
	≥ 50%	10 (27.8%)	5 (45.5%)	5 (20.0%)	0.116
	< 50%	26 (72.2%)	6 (54.5%)	20 (80.0%)	
%DLCO (%), median (IQR)*		60.5 (48.9, 68.1)	63.0 (55.9, 80.6)	58.5 (48.0, 67.0)	0.120
	≥ 55%	17 (65.4%)	9 (81.8%)	8 (53.3%)	0.132
	< 55%	9 (34.6%)	2 (18.2%)	7 (46.7%)	
pO_2_ (mmHg), median (IQR)		83.3 (72.8, 89.1)	84.3 (78.9, 96.1)	81.5 (71.0, 88.3)	0.159
	> 60 mmHg	33 (91.7%)	11 (100.0%)	22 (88.0%)	0.230
	≤ 60 mmHg	3 (8.3%)	0 (0.0%)	3 (12.0%)	
pCO_2_ (mmHg), median (IQR)		43.8 (40.5, 48.0)	41.7 (41.2, 44.1)	46.1 (39.8, 49.3)	0.257
	< 45 mmHg	20 (55.6%)	9 (81.8%)	11 (44.0%)	0.035
	≥ 45 mmHg	16 (44.4%)	2 (18.2%)	14 (56.0%)	
Survival	Alive (on waitlist)	3 (8.3%)	2 (18.2%)	1 (4.0%)	0.036
	Alive (received transplantation)	19 (52.8%)	8 (72.7%)	11 (44.0%)	
	Dead	14 (38.9%)	1 (9.1%)	13 (52.0%)	
APTD/TDT, median (IQR)		0.510 (0.481, 0.544)	-	-	-

APDT, anteroposterior diameter of the thoracic cage; BMI, body mass index; DLCO, diffusing capacity of the lung for carbon monoxide; FVC, forced vital capacity; GNRI, geriatric nutritional risk index; IQR, interquartile range; pCO_2_, partial pressure of carbon dioxide; pO_2_, partial pressure of oxygen; PPFE, pleuroparenchymal fibroelastosis; TDT, transverse diameter.

* Data were missing for 10 patients.

### Cut-off value of APDT/TDT

**Figure 1:**
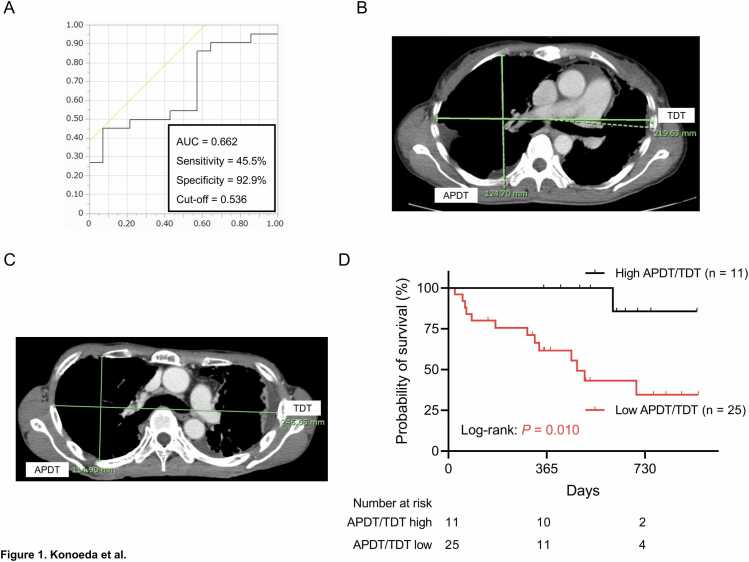
Prognostic significance of the APDT/TDT in patients with PPFE awaiting LT from DBD. (A) Receiver operating characteristic curve for APDT/TDT for prediction of waitlist mortality. Representative computed tomography images demonstrating (B) high and (C) low APDT/TDT values (0.568 and 0.466, respectively). (D) Kaplan–Meier analysis of waitlist mortality in the low APDT/TDT group (< 0.536; n = 25) and the high APDT/TDT group (≥ 0.536; n = 11) groups; log-rank: *P* = 0.010. APDT, anteroposterior diameter of the thoracic cage; DBD, donation after brain death; LT, lung transplantation; PPFE, pleuroparenchymal fibroelastosis; TDT, transverse diameter.

The median APDT/TDT was 0.510 (first, third IQR: 0.481, 0.544) (Table 1). ROC analysis for overall mortality identified an optimal APDT/TDT cut-off value of 0.536, with a sensitivity of 45.5%, specificity of 92.9%, and area under the curve of 0.662 (Figure 1A). Representative CT images illustrating high and low APDT/TDT values are shown in Figures 1B and 1C (0.568 and 0.466, respectively).

### Association between APDT/TDT and patient characteristics

On the basis of this cut-off, 11 patients (30.6%) were classified into the high APDT/TDT group and 25 (69.4%) into the low APDT/TDT group (Table 1). Compared with the high APDT/TDT group, the low APDT/TDT group had a shorter 6MWD (*P* = 0.002), lower %FVC (*P* = 0.015), and a higher proportion of patients with pCO_2_ ≥ 45 mmHg (*P* = 0.035). A history of pneumothorax was more frequent in the low APDT/TDT group, although this did not reach statistical significance (*P* = 0.058). LT was performed in 11 patients (44.0%) in the low APDT/TDT group and in 8 patients (72.7%) in the high APDT/TDT group, whereas 13 patients (52.0%) in the low group and 1 patient (9.1%) in the high group died during the waiting period (*P* = 0.036).

### Risk factors for waitlist mortality

The median follow-up duration was 467 days (first, third IQR: 298, 649). Median survival was significantly shorter in the low APDT/TDT group than in the high APDT/TDT group (478 days vs. not reached; *P* = 0.010; Figure 1D). One-year survival rates were 61.6% and 100.0%, and two-year survival rates were 34.5% and 85.7%, respectively. In univariate analyses, waitlist mortality was significantly associated with low APDT/TDT (HR 9.269, 95% CI 1.202–71.485; *P* = 0.033), a history of smoking (HR 4.586, 95%CI 1.399–15.035; *P* = 0.012), and 6MWD < 400 m (HR 5.017, 95%CI 1.549–16.254; *P* = 0.007) (Table 2). Although not statistically significant, low GNRI, higher %DLCO, and lower pO_2_ showed trends toward increased mortality (*P* = 0.092, 0.053, and 0.093, respectively).

**Table 2:** Results of univariate analyses of waitlist mortality in patients with PPFE patients listed for lung transplantation from donation after brain death

Variables	HR	95% CI	*P* value
Age (≥ 55 years/<55 years)	1.104	0.381–3.202	0.856
Sex (male/female)	2.590	0.720–9.322	0.145
Smoking history (yes/no)	4.586	1.399–15.035	0.012
BMI (< 20 kg/m^2^/≥ 20 kg/m^2^)	0.636	0.199–2.035	0.445
GNRI (low [< 93.84]/high [≥ 93.84])	3.002	0.835–10.802	0.092
6-minute walk distance (< 400 m/≥ 400 m)	5.017	1.549–16.254	0.007
Oxygen supplementation at rest (yes/no)	2.593	0.716–9.392	0.147
History of pneumothorax (yes/no)	2.042	0.706–5.909	0.188
Use of steroid (yes/no)	1.175	0.323–4.277	0.806
Use of antifibrotic agents (yes/no)	1.621	0.506–5.192	0.416
%FVC (< 50%/≥ 50%)	1.095	0.343–3.501	0.878
%DLCO (< 55%/≥ 55%)	3.757	0.981–14.388	0.053
pO2 (≤ 60 mmHg/> 60 mmHg)	3.909	0.796–19.184	0.093
pCO2 (≥ 45 mmHg/< 45 mmHg)	1.291	0.448–3.717	0.636
APDT/TDT (low [< 0.536]/high [≥ 0.536])	9.269	1.202–71.485	0.033

APDT, anteroposterior diameter of the thoracic cage; BMI, body mass index; CI, confidence interval; DLCO, diffusing capacity of the lung for carbon monoxide; FVC, forced vital capacity; GNRI, geriatric nutritional risk index; HR, hazard ratio; IQR, interquartile range; pCO_2_, partial pressure of carbon dioxide; pO_2_, partial pressure of oxygen; PPFE, pleuroparenchymal fibroelastosis; TDT, transverse diameter.

## Discussion

This study demonstrates that chest flattening, quantified by APDT/TDT, together with 6MWD, which is used to inform timing of referral to LT centers and listing for LT,^7^ significantly stratifies waitlist mortality in patients with PPFE awaiting LT from DBD. Notably, only 11 patients (44.0%) in the low APDT/TDT group ultimately underwent LT, compared with 8 patients (72.7%) in the high APDT/TDT group. In contrast, more than half of the patients in the low APDT/TDT group (n = 13; 52.0%) died while awaiting transplantation, with one- and two-year survival rates of only 61.6% and 34.5%, respectively. These findings suggest that chest flattening may present a clinically meaningful criterion for referral to LT centers and for listing, with the potential to reduce waitlist mortality and improve access to LT in patients with PPFE, although it is not currently incorporated into standard decision-making. In addition, living-donor LT may warrant more proactive consideration in patients with a low APDT/TDT.

The present study did not establish an optimal APDT/TDT threshold for referral or listing, and the cut-off value of 0.536 identified here may be too low for this purpose, given the markedly poor waitlist survival observed in the low APDT/TDT group. The association of low APDT/TDT with shorter 6MWD, lower %FVC, higher pCO2, and a history of pneumothorax further suggests that this cut-off reflects more advanced disease. In the light of prior work by Harada et al. demonstrating progressive worsening of chest flattening during the clinical course of PPFE,^4^ earlier referral to LT centers before substantial thoracic cage deformation occurs may be preferable. Identifying patients at risk for progressive chest flattening is therefore critical to selecting candidates who should undergo LT at the earliest appropriate time. Larger retrospective or prospective studies are needed to define an optimal APDT/TDT threshold for referral and listing.

Data on post-transplant outcomes in PPFE remain limited. Two nationwide multicenter studies, one from Japan and one from France, have evaluated LT outcomes in this population.^8^, ^9^ The Japanese study comparing 31 patients with idiopathic PPFE and 69 with idiopathic pulmonary fibrosis found comparable post-LT survival, although persistently reduced %FVC in PPFE was attributed to a rigid flattened chest.^8^ French study reported a high early mortality rate of 32% among 31 patients with PPFE, with a nonsignificant trend toward higher early mortality in those with a flat chest compared with those without (70% vs. 48%).^9^ These findings further support the potential benefit for performing LT before chest flattening becomes advanced.

Our previous nationwide study evaluating LT outcomes in patients with IPPFE and idiopathic pulmonary fibrotic (IPF) examined post-transplant changes in the thoracic cavity.^8^ In that study, the APDT/TDT ratio was significantly lower in the IPPFE group than in the IPF group. Additionally, among patients with IPPFE, the APDT/TDT ratio showed a statistically significant increase one year after LT from DBD (pre-transplant: 0.494 ± 0.07 vs. one year post-transplant: 0.521 ± 0.06; *P* < 0.01). However, the APDT/TDT ratio at one year post-LT in the IPPFE group remained significantly lower than that observed in the IPF group (IPPFE: 0.521 ± 0.06 vs. IPF: 0.583 ± 0.07; *P* < 0.01). The clinical implications of these post-transplant changes in thoracic morphology were not evaluated in that study and warrant future investigation in future research.

Several limitations associated with this study warrant mention. First, the small number of patients with PPFE limits the strength of the conclusions. Second, multivariate analysis was not feasible because of the limited number of deaths. Third, the median follow-up duration of 467 days was relatively short compared with the average LT waiting time in Japan of approximately 700 days.^10^ Fourth, because patients who underwent LT were censored at the time of transplantation, informative censoring may have been introduced, potentially resulting in bias in the survival analysis. Fifth, longitudinal data on changes in APDT/TDT prior to listing or during the waiting list, which could be relevant to waitlist mortality or post-transplant survival, were insufficient and therefore could not be analyzed. Sixth, as this study was conducted exclusively in Japan, where waiting times for LT are prolonged, the generalizability of the findings may be limited owing to differences in waiting periods and allocation systems across countries.

Collectively, these limitations underscore the need for larger registry-based investigations, such as the International Society for Heart and Lung Transplantation registry, to evaluate broader patient populations using appropriately powered and adapted statistical methods. Additionally, a multicenter prospective study exploring prognostic markers for waitlist mortality in patients, including those with PPFE, awaiting LT is currently ongoing in Japan. These investigations would clarify the true and definitive significance of flat chest in predicting waitlist mortality in patients with PPFE.

In conclusion, APDT /TDT, an index of a flat chest, emerged as a potential prognostic marker for waitlist mortality in patients with PPFE awaiting LT. Chest flattening may serve as a useful reference for timing of referral to LT centers, listing decisions, and determination of LT indication in this population.

## Ethical statement and consent for publication

This retrospective, descriptive, exploratory study of an ongoing cohort was approved by the Ethics Committee of The University of Tokyo Hospital (IRB#: 2406-(12); July 17, 2025). Owing to the retrospective design, study information was provided to all patients through an opt-out process, allowing them to decline the use of their data.

## Funding

The authors received no funding for this study.

## Declaration of Generative AI and AI-assisted technologies in the writing process

During the preparation of this work, the authors did not use generative AI.

## Declaration of Competing Interest

The authors declare that they have no known competing financial interests or personal relationships that could have appeared to influence the work reported in this paper.
